# The Association between Foveal Morphology and Macular Pigment Spatial Distribution: An Ethnicity Study

**DOI:** 10.1371/journal.pone.0169520

**Published:** 2017-01-09

**Authors:** Irene Ctori, Byki Huntjens

**Affiliations:** Applied Vision Research Centre, The Henry Wellcome Laboratories for Vision Sciences, City, University of London, London, United Kingdom; Universita degli Studi di Firenze, ITALY

## Abstract

**Purpose:**

Macular pigment (MP) spatial distribution varies considerably among individuals. We investigated ethnic variations in MP spatial distribution in relation to foveal architecture.

**Methods:**

We measured MP optical density (MPOD) using heterochromatic flicker photometry (MAP test, City, University of London) in 76 white, 80 South Asian and 70 black volunteers (18 to 39 years). MPOD spatial profiles were classified objectively as exponential, ring-like or central dip, based on deviations away from an exponential fit. Measurements including total retinal thickness (RT), inner retinal layer (IRL), inner and outer plexiform layer (IPL and OPL) thickness, foveal width and foveal pit slope were taken from Spectralis SD-OCT (Heidelberg, Germany) scans.

**Results:**

Integrated MPOD up to 1.8° (MPODint) was higher in South Asian (0.84±0.26) and black (0.84±0.31) than whites (0.63±0.24, P<0.0005). Ethnicity explained around 10% of the variance while gender played no significant role. MPOD profile phenotypes were associated with ethnicity: 58% with ring profiles were South Asian and 43% with dip profiles were black (χ^2^(4,226) = 13.4, P = 0.009). MPODint was lower in exponential (0.66±0.21) compared to ring-like (0.96±0.26) and central dip (1.00±0.32, P<0.0005) groups. White subjects had thicker IRL at 0° (130±21μm) than South Asian (123±16μm) and blacks (116±14μm; F(2) = 12.4, P<0.0005), with comparable results for IPL (P<0.0005) and OPL (P = 0.03). There was no significant difference in IRL, IPL or OPL (from 0 to 3.8° retinal eccentricity) or foveal width between MP profile groups (P>0.05).

**Conclusion:**

We report a significant difference in the amount and distribution of MP between ethnicities that is not explained by variations in foveal morphology.

## Introduction

Increased MPOD may reduce the likelihood of age-related macular degeneration (AMD) by protecting the retina from oxidative and photochemical damage [1]. MPOD has been shown to vary among individuals [2–5] and may be affected by age [6–9], gender [10], iris colour [3, 11] and modifiable factors such as smoking status [12]. MPOD typically follows an exponential decline with eccentricity from the centre of the fovea [13–16]. Previous studies have highlighted the occurrence of *atypical* MP spatial profiles also known as secondary sub-peaks [17], bi-modal [18], ring-like [19, 20] and central dip [21, 22] distributions. Increased prevalence of atypical ring-like profiles has been reported in females [18], non-smokers [21], healthy subjects compared to those with AMD [19] and in ethnicities that may present lower prevalence of AMD [22, 23]. However, there are inconsistencies in the literature regarding increased prevalence of ring-like profiles among non-white ethnicities [10, 14, 16]. Nonetheless, it has been shown that white subjects demonstrate lower central MPOD than non-whites, including South Asian [22, 24, 25] and black ethnicities [23].

A possible predictor of the MPOD spatial profile may be foveal morphology. Almost thirty years ago it was suggested that the spatial distribution of MP was attributable to its position within the individual retinal layers, with maximum concentration of MP in the fovea within the fibres of Henle and in the inner and outer plexiform layers in the parafovea [13, 26]. As the fibres of Henle extend horizontally into the inner nuclear layer, MP has been found between the cell nuclei of the nuclear layer [27] and there is evidence that the Müller cells interleaved amongst the cone photoreceptors also contain macular carotenoids [28, 29]. It has been suggested that variations in foveal pit morphology play a role in the spatial distribution of MP across the retina [16–18, 30]. In white subjects, a thicker central retina has been associated with significantly higher MPOD levels [30, 31]. However, such an association has not been reported in other studies [16, 17, 32]. It has been proposed that a sharper decline in MPOD with eccentricity from the fovea is associated with a steeper incline in retinal thickness from the centre of the fovea to the periphery, possibly due to compression of the inner plexiform and cone axon layers of the retina that host MP [17]. On the other hand, wider foveas support longer cone axons and may therefore provide more storage capacity for MP [16, 31].

Variations in foveal pit morphology have been found to vary across ethnicities [33–35], whereby non-white subjects have a significantly thinner central retina and wider foveas compared to whites [33, 35–39]. Given the inter-individual differences in both MPOD and foveal morphology, the current study was designed to investigate ethnic variations in MP spatial distribution in relation to foveal architecture with specific emphasis on the inner and outer plexiform layers.

## Methods

The investigation took place at the Division of Optometry and Visual Science, City University London between October 2013 and March 2015. Ethical approval for the study was obtained from the University Research & Ethics Committee and written informed consent was obtained from all subjects prior to participation, conforming to the tenets of the Declaration of Helsinki. Participants completed a health and lifestyle questionnaire, including information about general and ocular health, use of medication, nutritional supplementation, and smoking status. Exclusion criteria were visual acuity of 0.3 logMAR or worse in the test eye, ocular pathology in the test eye, previous refractive surgery, current use of carotenoid supplementation. Inclusion into the study was based on self-reported white, South Asian or black ethnicity and between 18 to 39 years of age. Classification of ethnicity was based on the criteria used by the Office of National Statistics [40]. The following ethnic groups were not included in the study: mixed/multiple ethnic groups; Asian Chinese or any other Asian background not mentioned in the inclusion criteria; and all other ethnic groups.

### Macular pigment measurements

MPOD was measured using the Macular Assessment Profile (MAP) test, a VDU-based test incorporating the HFP technique. Its rationale has been described in detail elsewhere [22, 41]. The MAP test measures MPOD at 0°, 0.8°, 1.8°, 2.8° and 3.8°. The average of two measurements at 6.8° and 7.8° retinal eccentricity serves as the peripheral reference point. As with other HFP methods, the luminance of the short wavelength test beam is altered to cancel or minimize the perception of flicker. A double reversal technique is employed to give the luminance of the test beam required to cancel the reference beam (the flicker null point) at each eccentricity. The average of four low and high threshold values is used to compute MPOD for each retinal location tested (calculated by comparing the mean luminance adjustment of the test beam in the central retina to the peripheral reference point). The area under the MP spatial profile curve from 0° to 1.8° i.e. MPODint (0 to 1.8) was calculated [17]. In addition, the slope of the MP spatial profile between 0° to 0.8°, 0.8° to 1.8° and 1.8° to 2.8° was calculated based on a previously described method [17].

### Foveal morphology measurements

Infrared scanning laser ophthalmoscope fundus imaging and SD-OCT (Spectralis, Heidelberg, Germany) imaging was performed on the test eye of each subject utilising the Automated Real Time eye-tracking feature. For each participant, mean keratometry and mean spherical error (MSE) measurements obtained using the TRK 1-P autorefractor (Topcon, Tokyo, Japan) were incorporated into the Spectralis SD-OCT software prior to scan acquisition to minimize errors in lateral measurements caused by ocular magnification [42, 43]. High resolution 20° x 10° volume scans (97 B-sections 30 microns apart, 16 frames including 1024 A-scans) were taken on an undilated eye in a dark room [16, 44]. Measurements of foveal morphology were obtained [45] and included:

- Total retinal, IRL, IPL and OPL thickness at eccentricities corresponding to the locations where MPOD is measured by the MAP test i.e. 0°, 0.8°, 1.8°, 2.8° and 3.8°,
- Total retinal volume derived from the 20° by 20° volume scan;
- Foveal width; and
- Foveal pit profile slope between 0° to 0.8°, 0.8° to 1.8° and 1.8° to 2.8°.

### Statistical analysis

All statistical analyses were performed using SPSS version 22.0 for Windows (SPSS Inc., Chicago, USA). Values in the text and tables are presented as the mean ± SD. Two-way ANCOVA evaluated the impact of ethnicity and gender confounders on MPOD and foveal morphology parameters. One-way ANCOVA was used to explore the effect of the MP spatial profile phenotype (exponential, ring-like or central dip) on foveal parameters. Pearson's product moment correlation coefficients were calculated to examine the association between retinal architecture parameters and MPOD measures. Statistical significance was accepted at the 95% confidence level (P < 0.05).

### Sample size calculation

An a priori power analysis conducted using G*Power 3.1 [46, 47] revealed that a total sample size of one hundred and ninety subjects was required. This was based on ANCOVA fixed effects, special, main effects and interactions calculated for three groups, with a power level of 80%, a statistical significance level of α = 0.05 and a medium effect size of 0.3.

## Results

In total, two hundred and twenty-six volunteers participated in the study, including seventy-six white (24 males; 52 females), eighty South Asian (31 males; 49 females) and seventy black subjects (25 males; 45 females). The right eye fulfilled the inclusion criteria and was therefore used as the test eye in two hundred and eighteen (96%) subjects. The range of MSE was -8.75 to +7.50DS in the white, -13.00 to +1.25DS in the South Asian and -7.75 to +1.75DS in the black ethnic groups. Mean MSE did not significantly vary between the three ethnic groups (P > 0.05). Thirty-two volunteers reported being current or ex-smokers (20 white, 7 South-Asian, and 5 black). Smoking pack/year was 0.98 ± 2.54 for the white, 0.043 ± 0.17 for the South Asian, and 0.048 ± 0.30 for the black ethnic groups. Due to the small number of smokers in the non-white groups, this variable was only considered within the white ethnic group.

### Variations in MP spatial distribution between ethnic groups

Mean central MPOD values were consistently lower in the white ethnic group (0.47 ± 0.17) compared to the South Asian (0.61 ± 0.17) and black ethnic groups (0.56 ± 0.19, P < 0.0005). This trend continued for MPOD at 0.8° and MPODint (0 to 1.8) as presented in Table 1. There were no statistically significant difference between the South Asian and black ethnic groups for any of these parameters (P > 0.05). While the main effect of gender reached statistical significance for MPOD at 0°, (F(1,226) = 4.63, P = 0.033), this was not the case for MPOD at 0.8° or MPODint (0 to 1.8) (P > 0.05). In any event the estimated effect size (partial eta squared) of gender was 0.02 or less on all MPOD variables.

**Table 1:** Mean ± SD MPOD at 0°, 0.8° and MPODint (0 to 1.8) per ethnic group and gender. Results of two-way analysis of covariance between-subjects effects of ethnicity are presented.

		White		South Asian		Black		P-value	Partial eta squared
		Mean	±SD	Mean	±SD	Mean	±SD		
**MPOD at 0°**	**Whole group**	0.47	0.17	0.61	0.17	0.56	0.19	**< 0.0005**	0.08
	Male	0.51	0.18	0.62	0.16	0.61	0.20	**0.033**	0.02
	Female	0.45	0.17	0.60	0.18	0.53	0.18		
**MPOD at 0.8°**	**Whole group**	0.39	0.16	0.53	0.17	0.52	0.20	**< 0.0005**	0.10
	Male	0.42	0.16	0.52	0.18	0.56	0.21	0.12	0.01
	Female	0.37	0.16	0.53	0.17	0.49	0.20		
**MPODint (0 to 1.8)**	**Whole group**	0.63	0.24	0.84	0.26	0.84	0.31	**< 0.0005**	0.11
	Male	0.68	0.24	0.84	0.27	0.92	0.32	0.06	0.02
	Female	0.61	0.23	0.83	0.25	0.79	0.29		

Among the white ethnic group, MPOD at 0° was higher in never smokers (0.49 ± 0.19) compared to current or ex-smokers (0.40 ± 0.12). However this failed to reach statistical significance (t(74) = 1.76, P = 0.08). Of note, the mean smoking pack/year ranged between 0 and 12 with a median < 0.0001. A multiple regression model explored the effect of retinal thickness and smoking on central MPOD in white subjects only. The model failed to reach significance (P = 0.08) and revealed that neither smoking (beta = -0.20) nor retinal thickness (beta = 0.17) made a unique contribution to MPOD.

**Fig 1:**
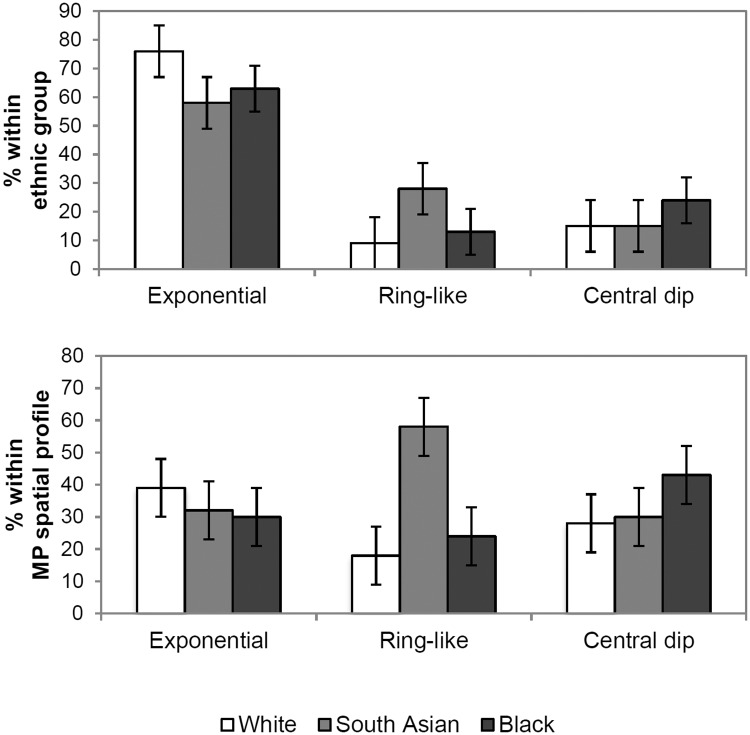
Percentage of individuals with exponential, ring-like or central dip MP spatial profile phenotypes within each ethnic group (upper graph). Percentage of white, South Asian and black individuals within each MP spatial profile phenotype (lower graph).

The percentage of individuals presenting each MP spatial profile phenotype within the three ethnic groups and within each MP spatial profile phenotype is provided in Fig 1A chi-square test for independence indicated a statistically significant association between ethnicity and presence of an exponential, ring or central dip MPOD spatial profile type (χ^2^ (4, n = 226) = 13.4, P = 0.009, Cramer's V = 0.17); the majority of subjects (58%) presenting with a ring-like MP spatial profile were of South Asian ethnicity, compared to 18% of white and 24% of black participants. Additionally more black subjects (43%) presented with a dip profile compared to whites (28%) or South Asian (30%).

### Variations in foveal architecture between ethnic groups

Mean ± SD foveal morphology parameters per ethnic group are presented in Table 2. A two way ANCOVA was conducted to investigate differences in foveal architecture between the white, South Asian and black ethnic groups, controlling for MSE. The main effects of ethnicity and gender were statistically significant for all foveal morphology dependent variables with a larger estimated effect size (partial eta squared) for ethnicity compared to gender.

**Table 2:** Mean ± SD retinal thickness, foveal width and foveal volume, inner and outer plexiform layers per ethnic group and results of two-way analysis of covariance showing between-subjects effects of ethnicity with mean spherical error as covariate (results for gender analysis not shown).

	White		South Asian		Black		P-value	Partial eta squared
	Mean	±SD	Mean	±SD	Mean	±SD		
**Retinal thickness at 0° (μm)**	229	20	220	14	215	14	**<0.0005**	**0.15**
**Inner Retinal Layer at 0° (μm)**	130	21	123	16	116	14	**<0.0005**	**0.12**
**Foveal width (μm)**	2282	225	2474	260	2449	284	**<0.0005**	**0.11**
**Foveal volume (μm**^**3**^**)**	8.86	0.34	8.71	0.35	8.73	0.39	**0.04**	**0.03**
**Inner plexiform layer (μm)**								
**at 0°**	13	3	12	3	11	3	**<0.0005**	**0.05**
**at 0.8°**	17	4	15	4	15	4	**<0.0005**	**0.11**
**at 1.8°**	31	6	26	6	29	6	**<0.0005**	**0.13**
**Outer plexiform layer (μm)**								
**at 0°**	5	5	5	5	3	3	**0.032**	**0.031**
**at 0.8°**	15	8	13	9	11	5	**0.003**	**0.051**
**at 1.8°**	30	6	28	9	27	10	0.264	0.012

Post-hoc Tukey testing indicated that IRL thickness was greater in whites compared to South Asian (P = 0.009) and blacks (P = 0.001). There was also a significant difference in IRL between South Asian and blacks (P = 0.009). These findings were replicated when the two-way ANCOVA was repeated for RT and IRL at 0.8° and 1.8°. Foveal width was increased in South Asian (P = 0.001) and blacks (P = 0.003) compared to whites. There was no significant difference between South Asian and blacks (P = 0.32).

The main effects of ethnicity and gender were statistically significant for IPL at 0°, 0.8° and 1.8°, with a small estimated effect size (partial eta squared < 0.1) for ethnicity and gender for IPL at 0° and a larger effect size for ethnicity and gender for IPL at 0.8° and 1.8°. Males had a tendency towards thicker IPL. Post hoc analysis revealed a thicker IPL in whites compared to the South Asian and black groups (P < 0.0005).

### Association of MPOD with foveal architecture according to ethnicity

For whole group analysis, there was no association between MPOD and RT at 0° (r = 0.12, P = 0.07). Following separate ethnic group analysis, there was no association in the white group (r = 0.16, P = 0.16) in comparison to South Asian r = 0.23, P = 0.04; black r = 0.34, P = 0.004). Although statistically significant for whole group analysis, the correlation between MPOD and IRL at 0° was weak (r = 0.18, P = 0.01). The strength of this association increased following separate ethnic group analysis (white r = 0.24, P = 0.03; South Asian r = 0.23, P = 0.04; black r = 0.38, P = 0.001). There were no statistically significant correlations between MPOD at 0.8° and 1.8° and corresponding retinal thickness (RT, IRL, IPL and OPL) for whole group or per ethnic group (P > 0.05 for all).

There were no significant correlations between MPOD at 0° and foveal width when analysed for the whole sample and per ethnic group (P > 0.05 for all). Likewise, MPODint (0 to 1.8) was not related to foveal width or volume (P > 0.05). In addition, there were no significant associations between the MP profile slope (0 to 0.8, 0.8 to 1.8 and 1.8 to 2.8) and corresponding foveal pit profile slope when the group was analysed as a whole or per ethnic group (P > 0.05). Gender appeared to play no significant role in any of our findings.

### Variations in foveal anatomy according to MP spatial profile phenotype

**Fig 2:**
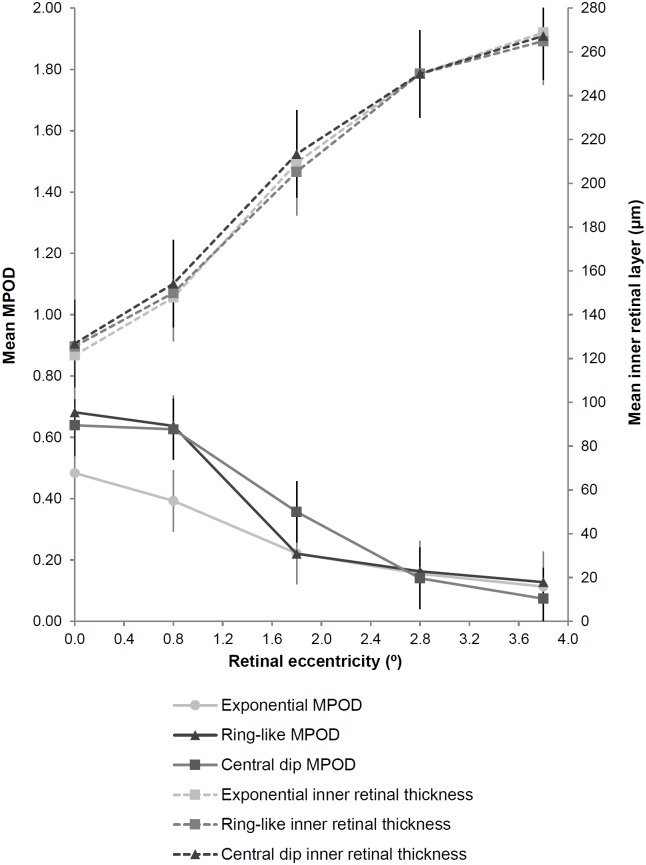
Variation in mean MPOD (primary y-axis) plotted against retinal eccentricity (x-axis) according to spatial profile phenotype with corresponding inner retinal layer thickness plotted on the secondary y-axis. Error bars indicate ±SD. Although MPOD at 0° and 0.8° is increased in the ring-like and central dip compared to the exponential spatial profile groups, there is no significant difference in inner retinal layer thickness between the groups.

We found no significant difference in retinal thickness (RT, IRL, IPL and OPL at 0°, 0.8° and 1.8°) according to MP spatial profile phenotype (irrespective of the ethnic grouping) (P > 0.05 for all). The lack of difference in IRL thickness between the three MP profile groups, in contrast to the significant variation in MPOD at 0° and 0.8° (P > 0.0005), is presented graphically Fig 2. On the other hand foveal width was significantly increased in the ring-like MP profile group (2516 ± 295μm) compared to exponential (2389 ± 267μm) and central dip profiles (2364 ± 270μm; F(2) = 4.28, P = 0.015). However, the estimated effect size was small (partial eta squared = 0.037). No difference in foveal volume was found between the three spatial profile groups (P = 0.78).

Investigation of association between MPOD and foveal anatomy variables per MP spatial profile group revealed a statistically significant moderate positive correlation between MPOD and total RT at 0° (r = 0.33, P = 0.04) and IRL at 0° (r = 0.42, P = 0.007) for the central dip group only. There were no other significant associations between MPOD at 0°, 0.8° and 1.8° and corresponding retinal thickness measures (RT, IRL, IPL and OPL); likewise there were no significant associations between MPOD at 0° and foveal width; or MPODint (0 to 1.8) and volume (P > 0.05). We also report a statistically significant moderate negative association between the MP profile slope and corresponding foveal pit profile slope between 0.8° to 1.8° (r = -0.40, P = 0.01), 1.8° to 2.8° (r = -0.33, P = 0.01) and 2.8) to 3.8° (r = -0.35, P = 0.01) for the central dip group only. This result indicates that a steeper decline in MPOD is associated with a shallower i.e. flatter foveal pit gradient for the central dip profile group.

## Discussion

The current study was conducted to investigate the association of MP spatial distribution and foveal architecture among young healthy white, South Asian and black subjects.

### Variations in MP spatial distribution between ethnic groups

White subjects, compared to South Asian and black subjects, presented with significantly lower levels of MP as represented by MPOD at 0°, 0.8° and MPODint (0 to 1.8). No statistically significant difference was found in these MPOD variables between the non-white ethnic groups. Around 10% of the variance in the MPOD measurements could be explained by ethnicity. In addition, our results suggest that non-exponential i.e. ring-like and central dip MP spatial profile phenotypes occur more frequently in individuals of South Asian and black ethnicity respectively, compared to white ethnicity (P = 0.009). This supports the suggestion that ethnicity plays a small role in the spatial distribution of MP [23, 48]. Whilst our findings support previous reports of a significant effect of gender of MPOD [10, 18], this was only apparent for MPOD at 0°. Smoking did not have a significant effect on MPOD; however, our study population only included a small number of smokers.

### Variations in foveal architecture between ethnic groups

We report that around 12–15% of the variation in retinal thickness was explained by ethnicity, while gender explained 3–5%. South Asian and black subjects presented with a statistically significant thinner central retina (RT and IRL) compared to whites, similar to earlier findings [35–39]. In contrast to a previous study [49] we found no significant difference in RT at 0° between the South Asian and black ethnic groups. Foveal width presented significant variations between the white and non-white ethnic groups in accordance with previous investigations that have shown that overall foveal morphology varies with fundus pigmentation [33, 50].

### Association of MPOD with foveal architecture according to ethnicity

A significant moderate positive association between MPOD at 0° and corresponding RT was determined, but this was only present in the South Asian and black ethnic groups. This is in agreement with the work by Nolan et al. whereby a lack of association between MPOD at 0.25° (measured by HFP) and averaged central foveal thickness in white subjects was found, compared to a significant positive correlation in a non-white sample that included South Asian, black and Hispanic subjects (r = 0.59, P < 0.01; n = 18) [16]. However, other studies have reported inconsistent findings; a positive correlation between central MPOD and central foveal thickness has been demonstrated [31], whereas others have found no correlation even when taking ethnicity into account [17, 31, 32]. In contrast, a significant negative correlation between central retinal thickness and MPOD at 0.5° (measured by HFP) was determined in a young healthy Caucasian cohort (r = -0.39, P = 0.01) [51]. The role of ethnicity remains unclear. Inconsistent reports of the correlation between MPOD at 0° with corresponding retinal thickness may be due to the location of MP specifically in the inner retina [13, 26, 29]. It is conceivable that any relationship that may exist between MPOD and retinal thickness is merely due to variations in IRL thickness whereby variations in total retinal thickness offset any underlying association. Indeed, we report a significant moderate positive relationship between MPOD and IRL at 0° demonstrated among the white, South Asian and black ethnic groups.

Interestingly, given that the non-white ethnic groups presented with thinner IRL thickness but presented with increased central and integrated MPOD, one might have expected to find a negative rather than a positive association between the two parameters. Rather than a linear association with retinal thickness, it has been proposed that the relationship between MPOD and corresponding retinal thickness may be governed by the overall shape or profile of the foveal dip created by thickness of the individual retinal layers beyond the foveola towards 2° eccentricity [52]. This finding could not be confirmed in the present study (Table 2). It therefore seems there is no immediate link between increased MPOD and increased thickness of the IPL or OPL. Furthermore, the hypothesis that a wider fovea is associated with increased MPOD was not supported by our findings.

### Variations in foveal anatomy according to MP spatial profile phenotype

We found that MP profile phenotypes were associated with ethnicity: 58% with ring profiles were South Asian and 43% with dip profiles were black (χ^2^(4,226) = 13.4, P = 0.009). In agreement with an earlier investigation [17], we found no significant variation in RT or IRL at 0° and 0.8° between subjects with exponential, ring-like or central dip MP spatial profiles. This suggests that increased retinal thickness is not responsible for the increased MPOD at 0° and 0.8° demonstrated in subjects with non-exponential MP spatial profiles. Inter-individual variations in the size and shape of the Müller cell cone may better explain the variations in MP distribution profiles. Indeed it has been postulated that the spatial arrangement of MPOD is created by the superimposition of the Henle fibre layer and the Müller cell cone [52]. Perhaps a monotonic decline of MP is due to a continuum of these structures whereby there is no superimposition of the Henle fibre layer and the Müller cell cone. Non-exponential profiles may therefore be a result of increased Müller cell cone thickness alone and a ring-like structure could be due to overlapping of the two structures.

While a positive correlation between the gradient of the MP and foveal pit has been reported [17], no association was established in a more recent investigation [52]. In the present study, no correlation between the MP profile slope and corresponding foveal pit profile slope was present for whole, ethnic or gender group testing. Interestingly, a significant negative association was present for the central dip group. Although the correlation was not strong, this finding suggests that a steeper decline in MPOD is associated with a shallower incline in the foveal pit profile slope. It would be of interest to apply more complex foveal pit modelling [53] to the current data to evaluate the sensitivity of the model to population demographic differences.

Our results imply that ethnic variations in MP and its spatial distribution cannot be explained by the differences observed in foveal morphology. Notwithstanding it is important to bear in mind that imaging of the retinal layers by SD-OCT is based on the optical properties of retinal tissue and the inbuilt algorithm to identify each layer. It has been proposed that the anatomical structures attributed to some of the hyper reflective bands may be incorrect and also may vary between devices [54]. Furthermore, not all retinal layers identified by histological studies are distinguishable on SD-OCT images. For example, the Henle layer has been visualised in vitro by histological examination [55, 56] but cannot be delineated by standard SD-OCT imaging (although a novel approach to achieve this has been described involving directionally altering the entry position of the SD-OCT beam through the subject's pupil [57, 58]). This in turn may explain the seeming lack of association of MP with foveal morphology.

## Conclusion

To our best knowledge this is the first study to consider the effect of ethnicity and gender on the association between MP and its spatial profile and foveal morphology. South Asian and black individuals presented with increased integrated MPOD, thinner central retinas and wider foveal pit compared to white individuals. Additionally, increased MPOD at 0.8° in ring-like profiles did not appear to be related to increased retinal thickness at its corresponding location. The results suggest that the spatial density distribution of MP is not a direct function of foveal morphology as measured in vivo by SD-OCT methods.

## Supporting Information

S1 File Data file for PloS ONE. (XLSX)
